# Evaluating impacts of improved flooring on enteric and parasitic infections in rural households in Kenya: study protocol for a cluster-randomised controlled trial

**DOI:** 10.1136/bmjopen-2024-090464

**Published:** 2025-06-06

**Authors:** Katherine E Halliday, Stella Kepha, Hugo Legge, Elizabeth Allen, Robert Dreibelbis, Lynne Elson, Beatrice K Kakoi, Carlos Mcharo, Sharon Muli, Jacinta Mwongeli, Doris Njomo, Margaret M Njoroge, Victoria Ochwal, William E Oswald, Martin Rono, Tuva K Safari, Ulrike Filinger, James Wambua Kaluli, Charles S Mwandawiro, Rachel L Pullan

**Affiliations:** 1 Department of Disease Control, Faculty of Infectious and Tropical Diseases, London School of Hygiene & Tropical Medicine, London, UK; 2 Eastern and Southern Africa Centre of International Parasite Control (ESACIPAC), Kenya Medical Research Institute, Nairobi, Kenya; 3 Department of Medical Statistics, Faculty of Epidemiology and Population Health, London School of Hygiene & Tropical Medicine, London, UK; 4 Kenya Medical Research Institute-Wellcome Trust Research Programme, Kilifi, Kenya; 5 Centre for Tropical Medicine and Global Health, Nuffield Department of Medicine, University of Oxford, Oxford, UK; 6 Department of Biomechanical and Environmental Engineering, Jomo Kenyatta University of Agriculture and Technology, Nairobi, Kenya; 7 Disease Ecology Group, International Centre for Insect Physiology and Ecology, Nairobi, Kenya; 8 College of Engineering and Technology, Jomo Kenyatta University of Agriculture and Technology, Nairobi, Kenya; 9 Global Health Division, International Development Group, RTI International, Research Triangle Park, North Carolina, USA

**Keywords:** Epidemiology, Public health, Randomized Controlled Trial, PARASITOLOGY, Gastrointestinal infections

## Abstract

**Introduction:**

Earthen floors are often damp or dusty and difficult to clean, providing an ideal environment for faecal pathogens and parasites. Observational studies have revealed associations between household flooring and health outcomes, but robust experimental evidence is scant. This study will evaluate the impact of an improved household flooring intervention on enteric infections, soil-transmitted helminth (STH) infections and tungiasis through implementation of a cluster-randomised trial in two rural settings in Kwale and Bungoma Counties, Kenya.

**Methods and analyses:**

440 clusters (households) across both sites are allocated to control or intervention group, in which a low-cost, sealed, washable, cement-based floor is installed in eligible buildings of the dwelling, alongside a floor-care guide provided during an induction meeting. Following baseline assessments in both groups, all individuals over 1 year receive albendazole and those infected with tungiasis receive benzyl benzoate. Primary outcomes are as follows: prevalence of enteric infections in children under 5 years assessed via stool surveys and PCR; prevalence of tungiasis infection in children 1–14 years based on clinical exam; and prevalence of STH infection in all household members over 1 year assessed via Kato-Katz. Secondary outcomes include the following: intensity of STH and tungiasis infections; prevalence of caregiver-reported gastrointestinal illness in children under 5; quality of life and well-being measures; and environmental contamination. A process evaluation investigates intervention acceptability, durability, practicality and cost.

**Ethics and dissemination:**

The protocol has been approved by ethics committees of The Kenya Medical Research Institute, The Kenya National Commission for Science Technology and Innovation, and The London School of Hygiene & Tropical Medicine. Following the 12-month implementation period and final assessments, control households are offered improved floors. Results will be disseminated within Kenya, to the Ministries of Health and of Lands, Public Works, Housing and Urban Development, and to subnational leadership and communities. Dissemination will also occur through publications and conference presentations.

**Trial registration number:**

NCT05914363.

**STRENGTHS AND LIMITATIONS OF THIS STUDY**

- The study has a robust two-arm, parallel, open-label, cluster-randomised trial design powered to detect differences across study arms for three primary health outcomes.
- Intervention and assessment methods are informed by extensive stakeholder engagement from local to national levels as well as in-depth formative work.
- The study includes a detailed process evaluation for analysis of the durability, feasibility and acceptability of the improved flooring intervention.
- Study follow-up is limited to 12 months, and so the study will not be able to evaluate longer-term durability, sustainability and impact of the flooring intervention.

## Introduction

 Access to adequate, safe and affordable housing is highlighted in the UN Sustainable Development Goals as vital to ensuring people can fulfil their potential in a healthy environment.^1^ In sub-Saharan Africa, upwards of 840 million people are estimated to live in inadequate housing.^2^ Structurally deficient housing can expose residents to health risks, including various infectious diseases.^3^ For example, earthen household flooring that is difficult to clean, and often damp or dusty, can provide an ideal environment for the survival of faecal pathogens and parasites.^4 5^ Observational studies (predominantly cross-sectional and cohort studies) repeatedly suggest that infections with diarrhoea-causing enteric pathogens such as *Giardia*, and *Cryptosporidium* spp., and STH are higher among individuals who live in houses with earthen floors.^5^,^12^ The home is also known to be a key transmission domain for parasitic sand fleas (*Tunga penetrans*) responsible for the inflammatory skin condition tungiasis, with research linking earthen flooring to increased prevalence.^13^,^15^ Combined, these conditions are responsible for considerable morbidity and mortality in rural communities,^16^,^18^ especially when local environmental contamination is high due to inadequate sanitation or living in close proximity to animals.^1419^,^21^

Despite the potential contribution of household flooring to the burden of enteric and parasitic infections in low- and middle-income settings,^5 22 23^ there have been few experimental studies involving flooring interventions. Nevertheless, from what is available, tangible benefits to health and well-being have been demonstrated. A feasibility study in Kenya found retrofitting a low-cost, cement-based floor into homes has the potential to reduce tungiasis infection intensity.^24^ An evaluation of a programme replacing earthen with cement floors in 34 000 households in Mexico revealed reductions of 19.6%, 12.8% and 20.1% for parasite infections, diarrhoea and anaemia, respectively, in children under 5 years, alongside improvements in cognitive development.^25^ A smaller-scale evaluation in Bangladesh has reported a 72% reduction in childhood diarrhoeal episodes associated with the installation of concrete floors.^26 27^ This limited evidence suggests that while flooring interventions may be able to deliver significant improvements in community health, there is a need for high-quality experimental data, specifically powered to assess relevant health outcomes, that can accurately and reliably quantify the impacts of such interventions.

This paper describes a cluster-randomised controlled trial (RCT) evaluating the impacts of an improved (cement-based) flooring and behaviour change intervention on enteric and parasitic infections in two settings in western and coastal Kenya. Intervention design was guided by mixed methods formative research,^23^ and an associated Theory of Change.^28^

### Aims and objectives

Our primary aim is to evaluate the effectiveness of an improved (cement-based) flooring intervention in reducing the burden of enteric infections, STH and tungiasis in participating households through implementation of an RCT in two distinct settings in rural Kenya.

The primary objectives are to quantify impact on the prevalence of enteric infections, STH infections and tungiasis. Secondary objectives include assessing impact on the intensity of STH infections and tungiasis, well-being of caregivers and children, self-report gastrointestinal illness in children and the extent to which entero-pathogen and parasitic contamination of floors is reduced within the home. We will also examine the differential effects across community and household contexts (including water, sanitation and hygiene (WASH) infrastructure, animal husbandry, user adherence and study site).

Finally, we deliver a process evaluation to determine the practicality, acceptability, costs and durability of the improved flooring intervention and how environmental, installation and use factors affect these.

## Methods and analysis

### Study design summary

The SABABU (Sakafu Bora Afya Bora - Utafiti; Kiswahili for Better Floors, Better Health - Research) trial is a two-arm parallel, cluster-randomised trial to measure the effect of the intervention 12 months post delivery. We consider households as clusters and, in each site, 220 households meeting the inclusion criteria are randomised (1:1) to either control or intervention arms. The intervention involves replacement of earthen floors with an improved cement-stabilised floor, accompanied by a behaviour change component (floor-care guide). In both study arms, all household residents over 1 year are offered a baseline treatment for STH (200 mg albendazole 12–23 months and 400 mg albendazole ≥24 months).

**Figure 1:**
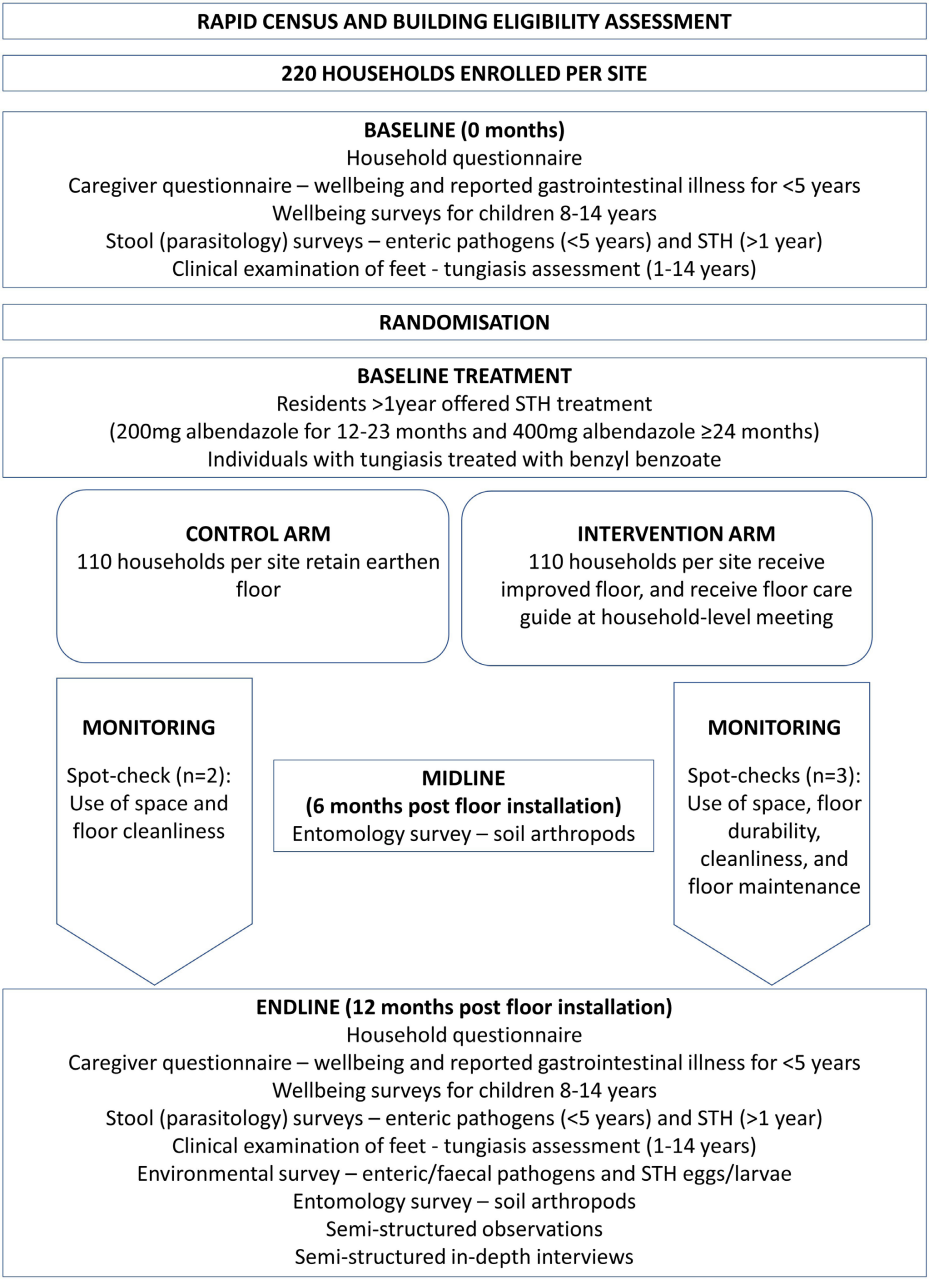
Study design. STH, soil-transmitted helminth.

In each household, prior to the STH treatment, stool samples are collected from all residents immediately before and 12 months post installation of floors (figure 1). Stool samples are assessed via PCR for enteric pathogens (children <5 years) and via Kato-Katz for STH (individuals >1 year), and clinical examinations are performed for tungiasis (children 1–14 years). During tungiasis assessments, those found to be affected are treated with benzyl benzoate (BB), provided by the study, at the point at which they are found, and the BB lotion is provided to the relevant community health volunteer to follow-up with two further treatments.^15 29^

In both study arms, quality of life measures in enrolled children and their caregivers are recorded immediately before and 12 months post installation of floors. Soil/dust sampling from all enrolled house floors is performed 6 months and 12 months post installation to screen for off-host *Tunga penetrans* stages. Environmental sampling for enteric pathogens is conducted on floors of a subsample of enrolled households at 12 months post installation. Alongside the trial, a process evaluation is undertaken to investigate intervention acceptability, durability, practicality and cost.

This protocol is reported in accordance with the Standard Protocol Items: Recommendations for Intervention Trials (SPIRIT) checklist (online supplemental file 1).

### Study settings

**Figure 2:**
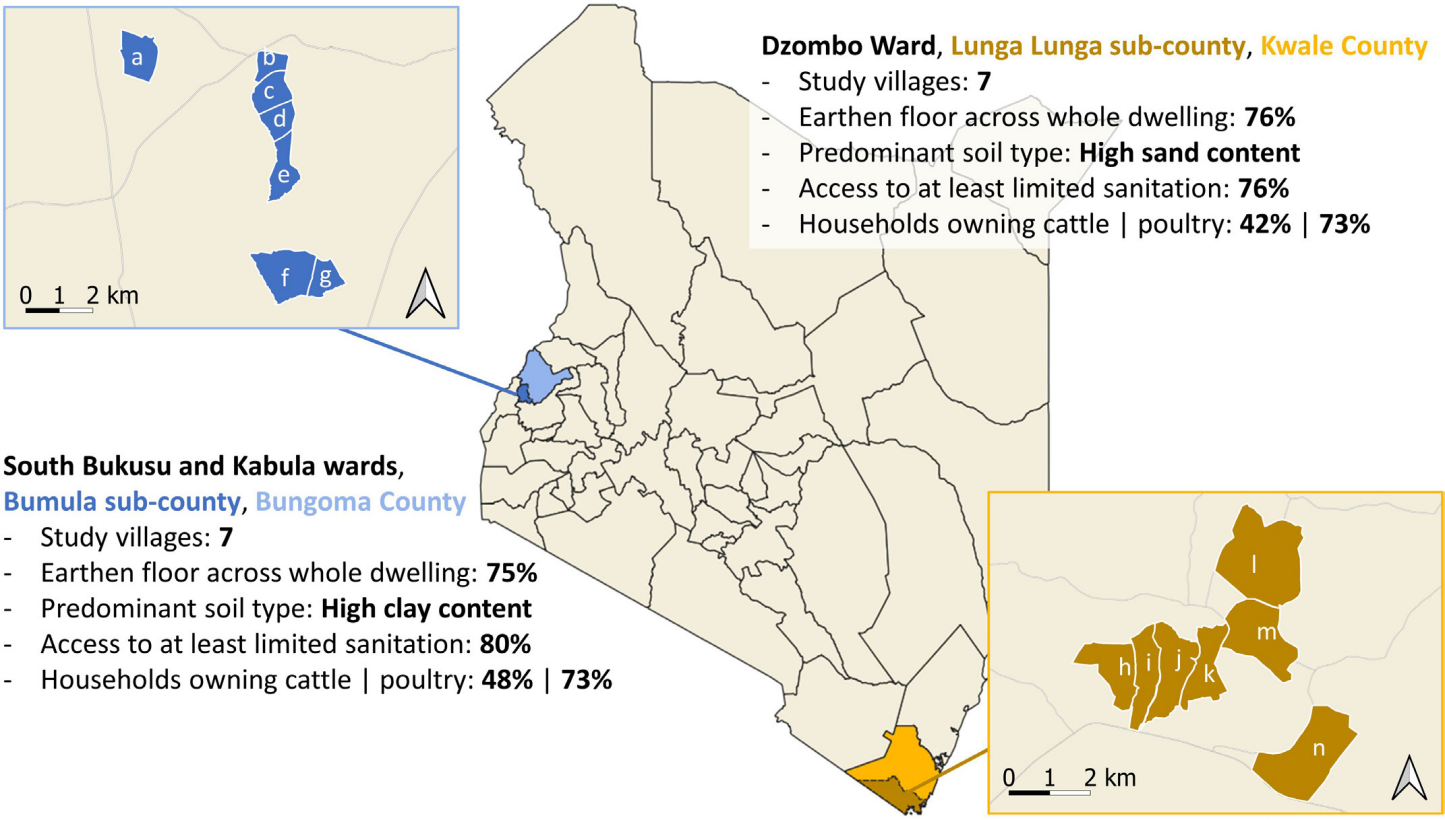
Map of study settings.

To improve generalisability and examine impact heterogeneity across environmental and cultural contexts, two sites are selected for the trial. Initially, formative work was carried out in 2021 in Kwale, Bungoma and Narok Counties to: identify housing conditions, map daily routines, and explore home improvement priorities to inform intervention development.^23^ Working with County Departments of Health, seven nearby villages per county were selected based on epidemiological profiles (reported STH and tungiasis endemicity) and housing conditions (high proportion of households with earthen floors). In Kwale and Bungoma, communities demonstrated a strong demand for sealable, washable floors, whereas in Narok this was less explicit with stakeholders citing other priorities. Moreover, a large majority of the traditional dwellings in Narok were deemed unsuitable for retrofitting of new floors. Subsequently, only Kwale County on the south Kenyan coast and Bungoma County in western Kenya (figure 2) are included in the trial.

Within Kwale County, the study villages are located in Dzombo ward, Lunga Lunga Sub-County. STH prevalence (predominantly hookworm) was 20% in 2017,^30^ reported diarrhoea prevalence in children under 5 was 3.2% in 2022^31 32^ and tungiasis prevalence has been found to be greater than 50% in some villages.^33^ Formative work in the seven villages censused 5241 individuals living across 812 households.^23^ 76% of house floors were earthen and 13% had unimproved (without slab) latrines. Within Bungoma County, the study villages span South Bukusu and Kabula wards in Bumula Sub-County. Prevalence of any STH in school-aged children is 7.3%,^34^ reported diarrhoea prevalence in children under 5 was 18% in 2022^31 32^ and tungiasis has been reported as present. Formative work among the seven study villages censused 4560 individuals living across 906 households, the majority of which (75%) had earthen floors and 16% of which had unimproved latrines.^23^

### Intervention

Our theory of change is described in detail elsewhere,^28^ with intervention design guided by three principles: ownership; community norms and standards; and awareness of existing routines. Briefly, installation and ongoing maintenance of improved household flooring are expected to reduce transmission of enteric and parasitic infections, by preventing direct exposure to pathogen and parasite contamination and through an intermediate effect of improved domestic hygiene. This is predicated on the assumptions that household members spend their time on the improved floor; it is a sealed surface limiting the opportunity for STH and sand flea off-host stages; it is washable and is regularly cleaned to reduce pathogen build-up and has limited animal contact to minimise faecal contamination. Improved household flooring is also intended to improve caregiver well-being through a number of routes including reduced health anxiety,^25^ improved comfort^35^ and increased levels of satisfaction, pride and self-efficacy.^36 37^ Child well-being could be impacted through increased energy levels resulting from decreased exposure to enteric pathogens, and reduced pain and itching as well as improved mobility, sleep patterns and concentration resulting from reduced exposure to skin parasites such as *Tunga penetrans*.^38^,^40^ The intervention consists of two components: (1) provision of a low-cost, sealed, washable floor throughout all eligible buildings in the dwelling and (2) a simple behaviour change component to support keeping these floors clean and well-maintained.

**Figure 3:**
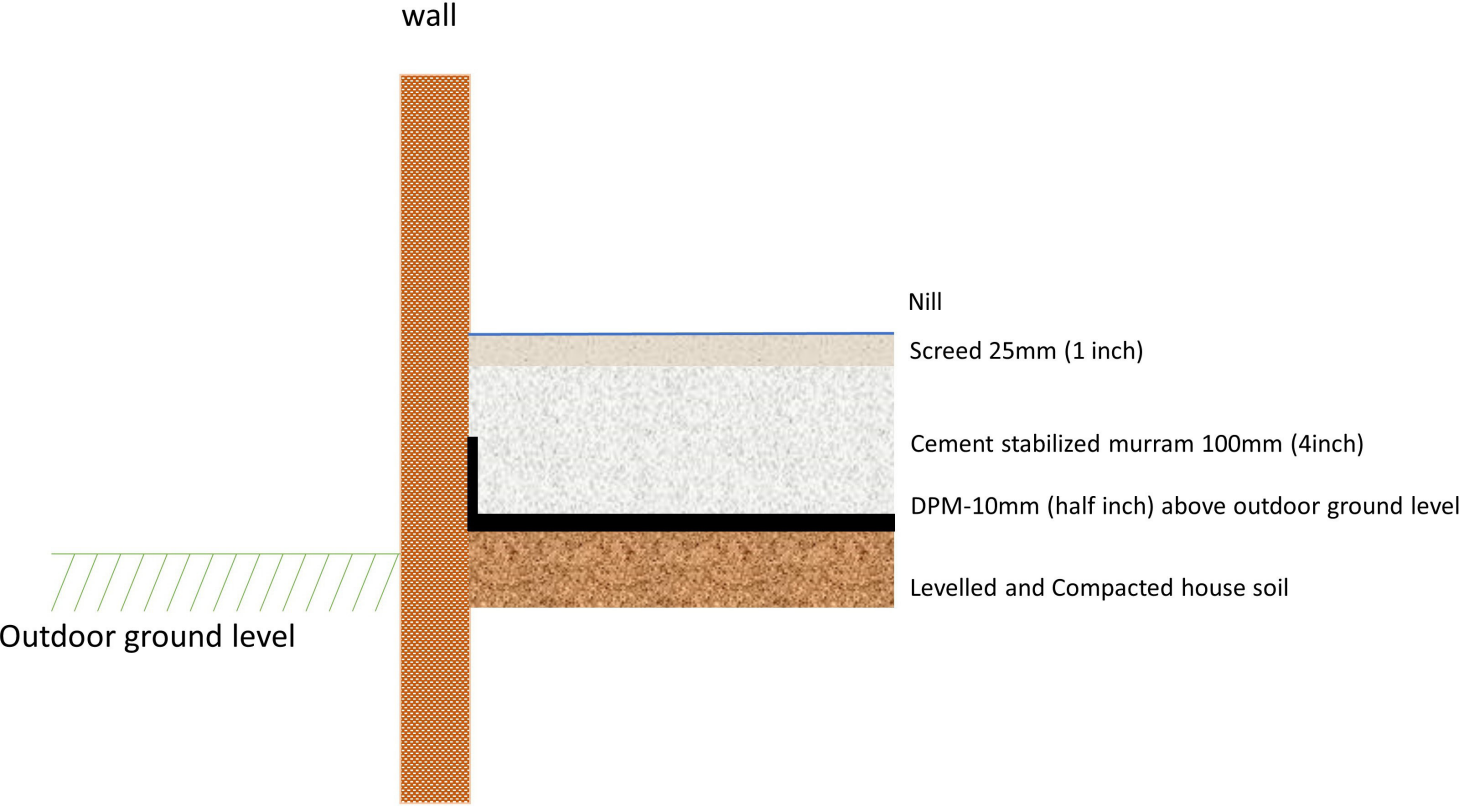
Technical drawing of the improved (cement-stabilised) floor installation.

A low-cost, cement-stabilised earthen floor is installed in each eligible building of the dwelling (excluding unimproved pit latrines, dedicated animal sheds and stores) to meet the following requirements: (1) non-absorbent, durable and smooth; (2) good wear resistance; (3) acceptable appearance; and (4) affordable. The decisions on flooring material and construction were informed by and built on a recent feasibility study exploring locally appropriate and affordable flooring solutions to create a sealed, washable floor for the prevention of tungiasis in coastal Kenya.^24^ The existing soil is broken up, levelled and compacted; a damp-proof plastic membrane is added to prevent dampness rising up through the stabilised floor; a cement-stabilised murram mix (1 part cement:9 parts murram) is added and compacted to a depth of 100 mm over the membrane; and finally, a screed mix (1 part cement:4 parts sand) is prepared and applied (25 mm), with a smooth cement and water slurry smeared on the screed to give a smooth finish (figure 3). During curing, water is sprinkled over the floor surface twice a day for 7 days.

All materials are provided by the study. Installation is performed by locally recruited masons, trained by the study team, with the support of local labourers. The study team provides day-to-day supervision and quality checks using a standardised checklist for all flooring activities, and the process is overseen by the same supervisors across sites. Additionally, to ensure consistency across sites, the masons from Bungoma travel to Kwale to observe the Kwale masons at work, and the Kwale masons travel to Bungoma to partake in the training and offer advice based on lessons learnt. Household members are not expected to contribute to labour or costs of laying floors but are required to vacate their dwellings for up to 9 days while floors are laid and cured. They are also requested to assist with water provision for the floor installation and conduct the curing process and are encouraged to contribute labour during excavation where practical.

The behaviour change component, developed in consultation with the community, comprises a floor-care guide and household-level floor-care meetings. The guide is a double-sided, laminated visual aid demonstrating the ‘dos’ and ‘don’ts’ of floor maintenance including regularly sweeping and washing the floor, avoiding fire directly on the floor, and keeping animals off, where possible (online supplemental file 2). Following floor installation (and before households move back into their dwelling), trained field officers visit to address any questions and to provide advice on floor-care.

### Trial outcomes

Primary outcomes are (1) enteric infections among children under 5; (2) tungiasis infection among children 1–14 years old; (3) and STH infection among residents of the same households who are at least 12 months of age; all of which are measured in both study arms at 0 months and 12 months (figure 1 and online supplemental file 2). A binary indicator of tungiasis infection is based on clinical examination and a binary indicator of any STH infection is assessed via Kato-Katz. For enteric infections, PCR of stool samples is used to identify prespecified enteric pathogens selected on the basis of a review of the literature:^59 41^,^43^ including *Giardia lamblia*, *Cryptosporidium* spp., *Campylobacter jejuni*, enteroaggregative *E. coli* (EAEC), *Shigella* and adenovirus, with the primary outcome based on infection with any pathogen versus none. Secondary outcomes include the following: caregiver-reported gastrointestinal illness in the last 7 days in children under 5 years; intensity of tungiasis and severity of tungiasis-associated pathology in children 1–14 years; well-being and quality of life in caregivers and children aged 8–14 years; enteric pathogen-specific prevalence among children under 5 years; and STH infection prevalence and intensity by species in individuals over 1 year.

To quantify reduction in contamination, secondary outcomes measured in both arms through environmental surveys of soil/dust samples include the following: contamination of floors with the same prespecified enteric pathogens and STH eggs/larvae (measured by PCR) and with eggs, larvae, pupae and adults of *T. penetrans* (measured using entomological screening).

Well-being in caregivers and children aged 8–14 years is measured using a range of standardised well-being and quality of life tools.

The differential effect of the intervention on the three primary outcomes (prevalence of enteric infections, STH and tungiasis) by context is examined via subgroup analysis stratified by study site and household contextual factors.

### Process evaluation

Formative research indicated potential for heterogeneity in how household members interact with the floor and adapt behaviours such as cooking, animal husbandry and sleeping, all of which may play an important role in mitigating intervention success.^23^ A process evaluation therefore explores implementation practicality, acceptability, durability and cost.

The practicality of retrofitting a floor and the consistency of delivery across sites are assessed using internal project reporting on the following: procurement, training, flooring installation, and implementation timelines. Installation quality checklists, observations and interviews with masons, study teams and households are also used. Consistency of the delivery of the behaviour change component is measured using internal project reporting, spot-checks and interviews with intervention households. The durability of the improved floor is observed in relation to environmental, installation and use factors at three structured spot-check visits, during which performance characteristics such as crazing, cracking and blistering are assessed by engineers.

Intervention acceptability is assessed through use of space within the dwelling measured preintervention and postintervention using self-reported measures, and the practice of target behaviours measured postintervention using spot-checks and direct semistructured observations. Caregiver satisfaction is measured using semistructured in-depth interviews and structured Likert scale questionnaires administered at endline to intervention households. Costs are measured using semistructured interviews and internal project records to assemble a cost breakdown for delivering each of the intervention components.

### Sample size calculation

Sample size and power calculations are based on primary outcomes and have been informed by data from Kenyan populations, including from the national school-based deworming programme,^34^ community-based tungiasis surveys^44^ and the Global Enteric Multicenter Study.^45^ Calculations are based on the principles of cluster-randomised trials, assuming an ICC of 0.1 based on small cluster size (household). Effect sizes for enteric pathogens are based on previous WASH efficacy studies.^46 47^ Effect sizes for tungiasis and STH are based on the research team’s expert opinion of the smallest meaningful public health effect. Tungiasis prevalence will be evaluated per site, while data on STH and enteric infections will be pooled across sites.

Expecting a postintervention STH infection prevalence of 15% in the control arm and 10% in the intervention arm, with an ICC of 0.1, and assuming five enrolled participants per household with a 15% loss to follow-up, 220 households per arm in total across the two sites provides 80% power at 0.05 significance. This sample size is also sufficient to detect at 80% power and 0.05 significance: (1) the expected difference in enteric infection risk in children <5 years old—assuming one <5 year old per household, and an expected prevalence postintervention of 70% in the control arm and 56% in the intervention arm; (2), the expected difference in tungiasis prevalence in children 1–14 years at a site level—assuming two children aged 1–14 per household and an expected prevalence postintervention of 30% in the control arm and 15% in the intervention arm. Based on these estimates, we aim to enrol 220 clusters (households) per arm—thus ensuring 220 children (aged under 5 years) and 440 children (aged 1–14 years) per arm across both sites.

### Sensitisation and engagement

Stakeholders at national, county and subcounty levels have been involved from conceptualisation. Prior to formative research, meetings were held at county, subcounty and ward levels (with village representatives) to introduce the study. Following formative research, consultative meetings were held at the ward and village levels, presenting research findings and compiling input on acceptability and ownership of the proposed intervention.^28^ This input informed development of care guides, and how to undertake engagement, given not all households would be eligible. Following selection of study villages and identification of eligible households, meetings are held with ward-level and village-level leadership to discuss plans for research participation and intervention roll-out. Additionally, preparation meetings are held with participants several days prior to the installation of the flooring intervention.

### Eligibility and enrolment

Inclusion criteria for households are as follows: at least one child under 5 years; home ownership; building where children under 5 sleep meets structural criteria (unimproved earthen flooring throughout, structurally sound); members providing informed consent; willing to temporarily relocate and provide water for installation. Exclusion criterion for households is as follows: intending to move within the next 12 months.

To assess eligibility, a two-stage census and eligibility assessment is conducted in study villages. Households are visited and a questionnaire is administered including questions on members’ sex, age and living arrangements, as well as direct observations of the dwelling’s building characteristics. Households meeting the core eligibility criteria are visited by a mason to assess the building for structural eligibility using a survey tool developed by structural engineers together with local masons. Eligible households are offered the opportunity to enrol and have baseline assessments undertaken.

### Randomisation

Site-level randomisation of households to control or intervention arms is stratified by village and done through a ceremony conducted in each site upon completion of baseline assessments. The ceremony is attended by members of the study team, village-level and ward-level leadership, and representatives from participating households, who each select a sealed card, indicating group A or B. Allocation to control or intervention arms is announced after all households have drawn cards.

### Timeline

The census and eligibility assessments were carried out in March 2023 in Kwale and June 2023 in Bungoma. Baseline assessments (0 months) are undertaken with all households prior to flooring installation in intervention households. Installation occurs following baseline assessments. Midline assessments are carried out 6 months post midpoint of floor installation. Three unannounced spot-checks are performed throughout the implementation period. Endline assessments are carried out 11–12 months after floor installation.

### Assessments

The assessments undertaken are described below and outlined in online supplemental file 2 . Once enrolled, any household which moves house is followed up in their new location, with a note made of this, providing they are still within the study sites.

A *household questionnaire* is administered to the primary caregiver at 0 and 12 months, including questions on demographics, assets, livestock and poultry ownership and husbandry practices, WASH infrastructure and practices, and building and room uses. WASH facilities and building conditions are directly observed and GPS coordinates of the dwelling collected.

A *caregiver questionnaire* conducted with primary caregivers at 0 and 12 months includes prevalidated question modules based on the WHO Five Well-being Index (WHO-5)^48^ and WHO Quality of Life BREF (WHOQOL-BREF)^49 50^ to evaluate respondent psychological well-being, and to ask about episodes of gastrointestinal illness in the last 7 days for all children under 5 years.

A *child questionnaire* is conducted with children aged 8–14 years at 0 and 12 months comprising questions about psychological well-being and perceived quality of life drawn from the WHO-5 and the EuroQol 5-Dimension Health-related Quality of Life for Youth (EQ-5D-Y) tools.^51 52^ The modified dermatological quality of life index for tungiasis is administered to children with tungiasis.^17 53^

During the *stool survey*, all household members are invited to provide a single stool sample at 0 and 12 months for screening for enteric infections (children under 5 years) and STH infections (all residents over 1 year). Participants are provided with sample collection kits and instructed how to collect an early morning sample for collection and transport to the laboratory by the study team.

A *tungiasis assessment* is conducted with children aged 1–14 years, supported by a parent/guardian if appropriate, at 0 and 12 months by trained field workers. Diagnosis of tungiasis is made on the basis of the presence of embedded fleas and manipulated lesions in participant’s feet. The viability and number of lesions are recorded.

*Entomological sweeps* undertaken at 6 and 12 months include collection of sand, dust and fine debris from floors in three or more locations in intervention and control households: (1) food preparation areas, (2) sitting/resting locations indoors and/or outdoors, and (3) child sleeping areas. Improved floors are swept and dust removed from crevices and gathered into zip-lock bags. Soil from unimproved floors is gathered with small brushes or trowels. Zip-lock bags are shipped in cool-boxes to the laboratory.

*Environmental surveys* examine for the presence of enteric pathogens and STH at 12 months. Floor dust samples are collected in three locations within dwellings (75 per arm per site): (1) 50cm in from the exterior entrance to the primary building, (2) 50 cm inside the room used for cooking, and (3) 50 cm inside the room where the youngest child sleeps. Adapted from soil sampling protocols,^19 54 55^ study personnel demarcate surface areas using a sterilised 50×50 cm stencil and sweep the enclosed surface with a sterilised paintbrush. This is repeated until, at each location, 2 g dust has been collected into sterile 2 mL sample collection tubes.

*Unannounced* *spot-checks* are performed at three points during the implementation period in all intervention households. A structured checklist is used to assess the following: floor structural condition (cracking, abrasion, water resistance); levels of debris; and location and duration of daily activities. An adapted checklist is conducted twice in control households, assessing levels of debris and location and duration of daily activities.

*Semistructured* *observations* are undertaken in 20 intervention and 20 control households in each study site at 12 months to quantify time spent by children, caregivers and animals in different parts of the dwelling and to identify where different activities are undertaken.

*In-depth* *interviews* are conducted at 12 months with caregivers from a random sample of intervention houses in which observations are performed (12 per study site). These cover user satisfaction with the floor, cleaning regimens, animal husbandry practices, food preparation, relaxation, child caregiving and perceptions on social status and the economic value of the floor. Interviews take 30–60 min and are conducted by trained interviewers using prepiloted question guides.

*Cost data collection:* Cost data are collected using an ingredients approach and a standardised costing framework, based on semistructured questionnaires and consultation of the project accounts.

### Laboratory work

Stool samples from children <5 years are aliquoted into cryovials for storage in 95% ethanol at −80°C. DNA is extracted from the stool using the QIAGEN QIAamp DNA Mini Kit^56^ and real-time quantitative PCR is used for the detection of prespecified enteric pathogens: *G. lamblia*, *Cryptosporidium* spp., *C. jejuni*, enteroaggregative *E. coli* (EAEC), *Shigella* and adenovirus.

Stool samples from individuals >1 year are processed and examined in duplicate (41.7 mg template) for presence and quantity of STH eggs using the Kato-Katz method.^57^ Duplicate slides are prepared from the single day stool samples and are read by independent microscopists with a 10% quality control check performed.

Samples collected during the entomology sweeps undergo heat extraction of soil arthropods using a Berlese-Tullgren apparatus.^58 59^ The presence and abundance of eggs, larvae, pupae and adults *T. penetrans* and other flea species as well as other arthropods, such as bedbugs, are recorded using a stereo microscope.

DNA is extracted from soil samples collected during the environmental surveys according to specifications outlined in the QIAGEN DNeasy PowerSoil Pro documentation (QIAGEN, Hilden, Germany) and is assessed for the preselected enteric pathogens and STH egg/larvae DNA using the established real-time PCR panel.

### Patient and public involvement

Intervention design, including logistics of fitting floors and development of floor-care guides, was shaped by findings from formative research and direct feedback from community members during community engagement activities. Floors are installed by local masons from study communities, with participant households providing water for curing. Community members provided feedback into the range of assessment methodologies used and proposed for the trial.

## Statistical considerations and analyses

### Data management

Quantitative data are primarily collected and stored electronically via SurveyCTO using encrypted Android tablets, and downloaded and transferred via an encrypted connection to a secure server. Interviews are conducted in person and audio-recorded. Audio files are digitally transcribed with identifiers removed and destroyed once transcriptions are complete. All electronic field notes, anonymised interview audio-recordings, and transcripts are stored on a secure server. Paper field notes are kept in a locked cabinet. Photographs are stored on a secure server for up to 5 years before being deleted.

### Statistical analyses

Analysis of primary and secondary human infection outcomes will be carried out on groups as randomised (intention-to-treat). Results will be presented as appropriate effect sizes (risk ratios for the primary outcomes) with a measure of precision (95% CIs), using generalised linear models with robust SE to account for correlation of outcomes within households. Prespecified analyses of impact heterogeneity will be conducted through the inclusion of interaction terms for household WASH access, ownership of livestock and socio-economic status.

Analysis of the extent to which the intervention reduces entero-pathogen and parasitic contamination of floors within the home will use generalised linear models with robust SE to estimate differences in overall pathogen prevalence in the dwelling environment at endline.

Generalised linear models with robust SE will be used to estimate differences in WHO-5 well-being index, WHOQOL-BREF (caregivers only) and EQ-5D-Y (children only) scores between study arms. Results will be triangulated with data from in-depth interviews with caregivers, which will be used to explore the pathways through which caregiver and child daily routines have changed, and if these changes have impacted well-being. Data will be coded deductively using preidentified themes and inductive coding will allow new themes to be identified while reading and interpreting transcripts. Observation data will be analysed thematically based on a case-memo approach.

Cost data will be used to estimate financial and economic costs of the intervention. Itemised costing and sensitivity analysis will enable estimation of the costs of scaled-up implementation.

## Ethics and dissemination

### Ethical considerations

The protocol was reviewed and approved by The Kenya Medical Research Institute (KEMRI) Scientific Ethics Review Unit (SERU) (05/06/424/4632), The Kenya National Commission for Science Technology and Innovation (NACOSTI) Review Board (NACOSTI/P/21/9594) and The London School of Hygiene & Tropical Medicine (LSHTM) Ethics Review Committee (28307).

Prior to starting any research activity, Swahili information sheets are provided and written informed consent (participants 18 years or older and parents/guardians of participants) and, where appropriate, written assent (children aged 13–17) or verbal assent (children aged 7–12) are sought. Written informed consent is sought from the household head for activities pertaining to the household (online supplemental file 2). Participants who are unable to sign apply a thumbprint using an inkpad, having identified a literate impartial witness who sits through the consenting process and additionally signs their own signature on the consent form. To safeguard participants during clinical and well-being assessments, trained team members conduct examinations and interviews in private locations with a second trained member chaperoning. Study data are handled according to the guiding principles for ethical research of partner institutions as well as MRC Guidelines for Good Clinical Practice in clinical trials.

Risks and benefits of participation are presented during community meetings and issues arising discussed. After the final assessments at 12 months, all control households are offered an improved floor. Retrofitting floors involves making substantial irreversible changes to dwellings, so participants are asked to consider carefully before agreeing to take part. Precautions are made to only enrol households in structurally suitable dwellings; however, in the potential event of damage as a direct result of the installation process, the study will be responsible for repairs. If it is not clear whether the installation process is directly responsible for any perceived damage, an independent engineer will be consulted. All tungiasis cases detected at baseline and endline are treated with BB lotion, provided by the study, and the BB lotion is provided to the relevant community health volunteer to follow-up with two further treatments.^15 29^ All household members over 1 year are offered STH treatment after our assessments at baseline and endline.

### Dissemination

Findings from this research will improve our understanding of important social determinants of health, helping guide environmental health priorities in Kenya and beyond. Results will be proactively disseminated to community-level, subcounty-level and county-level leadership. It is intended that the results will inform development of future public health and housing policy within Kenya, and as such, results will be communicated to the Ministry of Health and Ministry of Lands, Public Works, Housing and Urban Development. Dissemination will take place beyond Kenya through presentations at academic conferences and publication in peer-reviewed journals, with the intention that other countries with similar housing and epidemiological profiles will be able to benefit.

## Supplementary material

10.1136/bmjopen-2024-090464 online supplemental file 1

10.1136/bmjopen-2024-090464 online supplemental file 2
