# Supplementary material for: Evaluating impacts of improved flooring on enteric and parasitic infections in rural households in Kenya: study protocol for a cluster-randomised controlled trial
Source: BMJ Open. 2025 Jun 6;15(6):e090464. doi: 10.1136/bmjopen-2024-090464 (PMC12161379; doi:10.1136/bmjopen-2024-090464)
Supplement: online supplemental file 2 [file bmjopen-15-6-s002.pdf]

Summary of aims, objectives, outcomes, measurement and sampling approach

Aims	Objectives	Outcomes	Who / What	Measurement	When	Which	Estimate sample numbers per site (total)
Aim 1: To evaluate the effectiveness of an improved flooring intervention in reducing the burden of enteric infections, STH and tungiasis in participating households through implementation of an RCT in two distinct settings in rural Kenya	**To quantify the impact of the intervention on prevalence of enteric infections, STH and tungiasis**	Prevalence of enteric infections	Children <5 years	Stool survey and real-time (RT)-qPCR	0 and 12 months	Control and intervention arms	220 (440)
		Prevalence of tungiasis infection	Children 1-14 years	Tungiasis clinical assessment	0 and 12 months	Control and intervention arms	600 (1200)
		Prevalence of at least one STH infection	All household members >1 year	Stool survey and Kato Katz microscopy	0 and 12 months	Control and intervention arms	1300 (2600)
		Prevalence of gastrointestinal illness	Children <5 years	Caregiver-reported symptoms via caregiver questionnaire	0 and 12 months	Control and intervention arms	220 (440)
		Tungiasis intensity	Children 1-14 years with tungiasis	Tungiasis clinical assessment	0 and 12 months	Control and intervention arms	Prevalence-dependent
		STH infection prevalence and intensity by species	All household members >1 year	Stool survey and Kato Katz microscopy	0 and 12 months	Control and intervention arms	Prevalence-dependent
	**To quantify the extent to which the intervention reduces entero-pathogen and parasitic contamination of floors within the home**	Environmental contamination for human-specific and animal faecal markers and STH eggs/larvae	Household dwelling n=3 samples per dwelling	Environmental survey and RT-qPCR for soil samples n=150 houses per site	12 months	Control and intervention arms	450 (900)
		Contamination of floors with eggs, larvae, pupae and adults of *T. penetrans*	Household dwelling n=3 samples per dwelling	Entomology survey, heat extraction and microscopy	6 and 12 months	Control and intervention arms	660 (1320)
	**To examine the effect of the intervention on the wellbeing of caregivers and children**	Wellbeing	Caregivers and children aged 8-14 years	WHO-5, WHOQOL-BREF, and EQ-5D-Y wellbeing and quality of life tools	0 and 12 months	Control and intervention arms	520 (1040)
		Tungiasis-related quality of Life	Children 8-14 years with tungiasis	Modified dermatological quality of life index for tungiasis	0 and 12 months	Control and intervention arms	Prevalence-dependent
	**To assess the differential effects of the intervention across community and household contexts (including site, WASH infrastructure, animal husbandry, user adherence)**	Prevalence of enteric infections stratified by study site and household contextual factors	Children <5 years	Subgroup analysis of the prevalence of enteric infections	0 and 12 months	Control and intervention arms	220 (440)
		Prevalence of tungiasis infection stratified by study site and household contextual factors.	Children 1-14 years	Subgroup analysis of the prevalence of tungiasis infection	0 and 12 months	Control and intervention arms	600 (1200)
		Prevalence of at least one STH infection stratified by study site and household contextual factors	All household members >1 year	Subgroup analysis of the prevalence of STH infections	0 and 12 months	Control and intervention arms	1300 (2600)

	**To examine the extent to which the intervention is practical and delivered consistently across both study sites**	Delivery of i) training and equipping masons, and ii) retrofitting the flooring infrastructure	Core research team and masons	Direct semi-structured observations and installation quality checklists.	Point of delivery of intervention	Intervention arm	110 (220)
**Aim 2:** To determine the practicality, acceptability, durability, and cost of an improved flooring intervention in two distinct settings in rural Kenya through delivery of a process evaluation		Delivery of the behaviour change components of the study	Research supervisors	Internal project reporting of floor care guide handover	Point of delivery of intervention	Intervention arm	110 (220)
	**To determine the acceptability of the intervention to participants**	Use of space within the dwelling by household members and animals	Caregivers	Self-reported use of space questionnaire.<br>Semi-structured observations n=20 per arm per site	3 timepoints<br>12 months	Control and intervention arms	220 (440)<br>40 (80)
		Practicing of target behaviours by household members	Caregivers	Structured spot-checks<br>Semi-structured observations n=20 per arm per site	3 timepoints<br>12 months	Control and intervention arms	220 (440)<br>40 (80)
		Caregiver satisfaction with the intervention	Caregivers	Semi-structured in-depth interviews<br>Likert-scale questionnaires	0 and 12 months<br>12 months	Intervention arm	12 (24)<br>110 (220)
	**To examine the durability of the improved floor and how environmental, installation, and use factors affect this**	Performance characteristics (strength) of the installed floors (absence of cracks, blisters, crazing and spalling)	Household dwelling	Structured spot-checks	3 timepoints	Intervention arm	110 (220)
	**To determine the major cost drivers of the intervention**	Cost of intervention	Core research team	Cost breakdown for delivering each of the intervention components	Continuous throughout implementation	Intervention arm	-

Improved Flooring Care Guide (English)

Clean floors keep your family healthy.

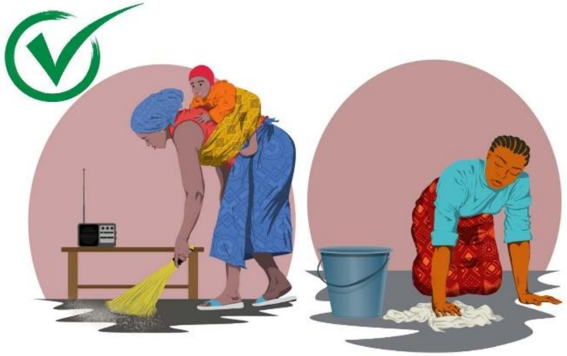

- Sweep the floor at least once a day
- Mop the floor with water and soap at least once every two days

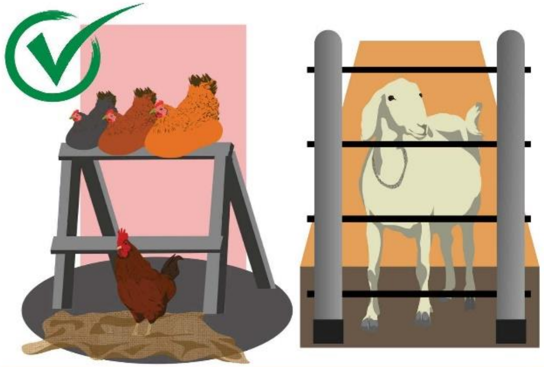

- Keep animals away from the floor
- If you can, find somewhere else for your animals to sleep

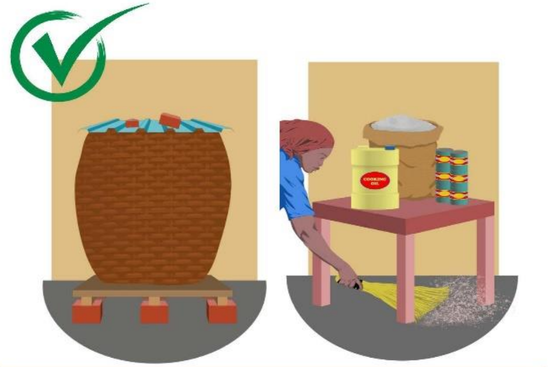

- Find ways to keep bulky items raised up to make it easier to clean the whole floor

Take care of your floor, it will last longer.

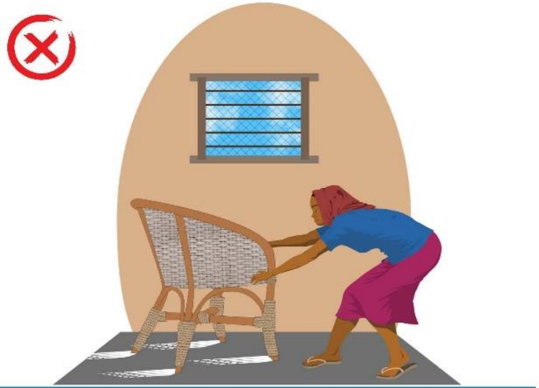

- Do not drop or drag heavy items on the floor as this can damage the floor

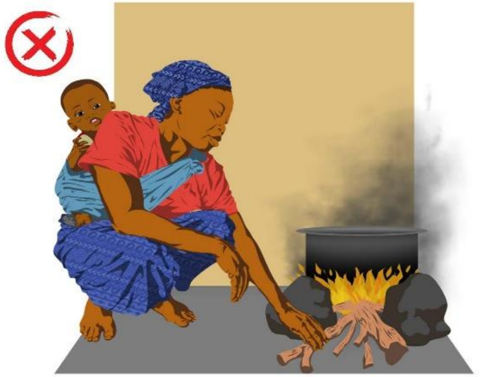

- Avoid direct fires on the floor as this can damage the floor

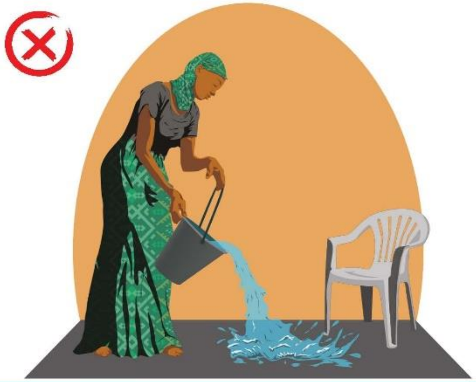

- Avoid flooding the floor with water when cleaning as this can damage the floor

Images credit: Curtis Luwembe

Improved Flooring Care Guide (Kiswahili)

Sakafu safi hudumisha afya ya familia yako

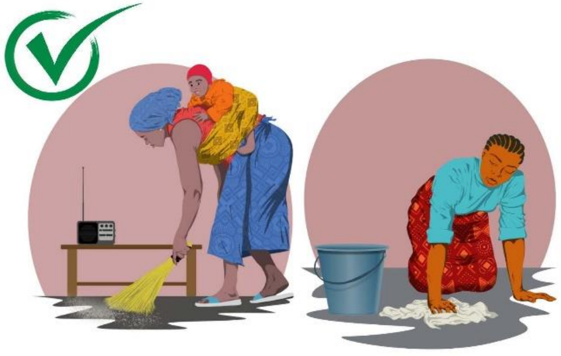

- Fagia sakafu angalau mara moja kwa siku
- Safisha sakafu na maji anagalau mara moja kila siku mbili

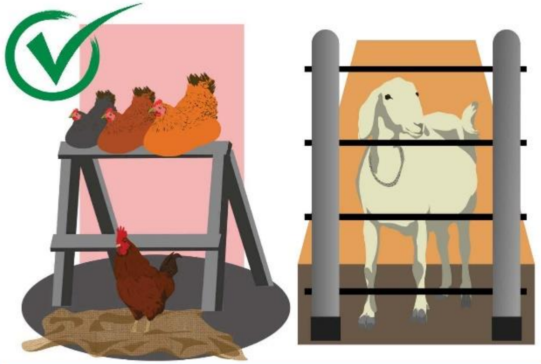

- Ondoa/weka wanyama mbali/kutoka kwa sakafu
- Panapowezekana tengeneza makazi mbadala kwa wanyama

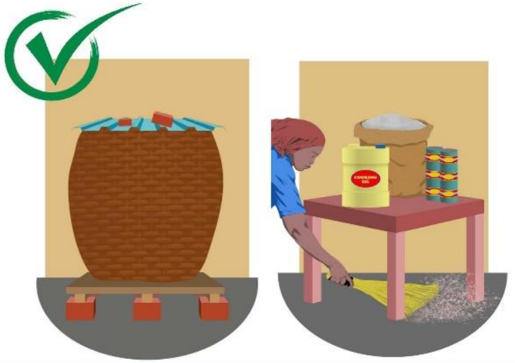

- Tafuta njia za kuhifadhi vitu kwa kuviinua juu zisilalie sakafu, ili iwe rahisi kusafisha sakafu nzima

Tunza sakafu yako itadumu kwa muda mrefu

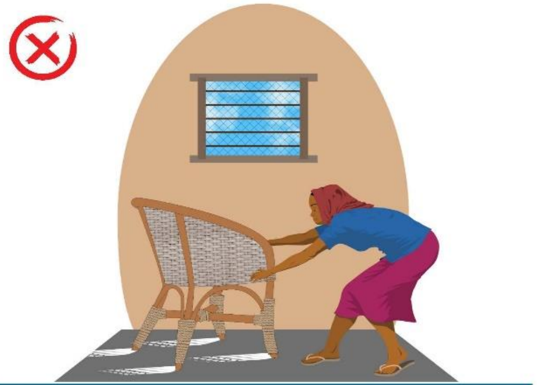

- Usiangushe wala kuburuta vitu vizito kwenye sakafu, hii inaweza kuharibu sakafu

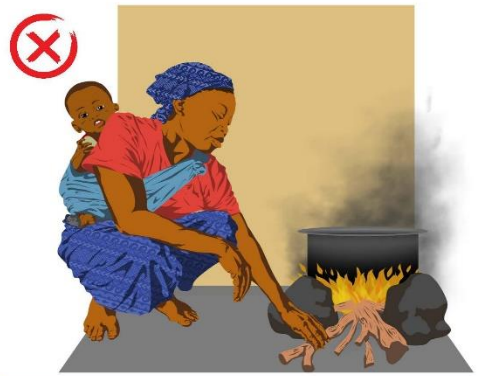

- Epuka kuwasha moto moja kwa moja kwenye sakafu hii inaweza kuharibu sakafu

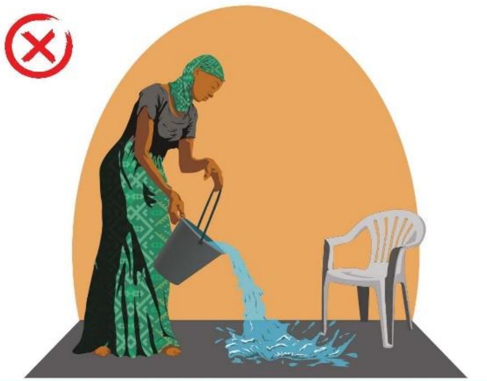

- Epuka kumwaga maji mengi kwenye sakafu wakati wa kusafisha, hii inaweza kuharibu sakafu

Images credit: Curtis Luwembe

Model Information sheet and Consent form

Annex 2: study enrolment information sheet and consent form for heads of household

Information Sheet 201: household study enrolment

Understanding the contribution of household flooring to disease burden in rural Kenya

Introduction

We would like to invite you to participate in a research study about households floors. Joining the study is entirely up to you. Before you decide, you need to understand why the research is being done and what it would involve. I will read and give you a copy of this information sheet and go through it with you, and answer any questions you may have. If you agree to take part, we will then ask you to sign a consent form. This study is being conducted by KEMRI in collaboration with its partners; the London School of Hygiene & Tropical Medicine (LSHTM); the Jomo Kenyatta University of Agriculture & Technology (JKUAT), and; the International Centre of Insect Physiology & Ecology (icipe).

What is KEMRI

KEMRI is a government organisation that carries out medical research. Research is different from normal treatment because research aims to find better ways of preventing and treating illness in the future for everybody's benefit. In this study KEMRI is working with other organisations which are; LSHTM – a university in the United Kingdom that also conducts medical research; JKUAT – a university in Kenya that does engineering research; icipe – a research institute in Kenya that explores how insects can affect people's health.

What is the purpose of the study?

Earthen floors are difficult to clean and often damp, providing a good environment for germs and parasites that may make you sick. To investigate whether household floors play a role in the spread of intestinal worms, diarrhoeal disease and jiggers, this study will run a research study in your community to assess if households with cement-based floors are affected less by these diseases. To undertake this study we will install new cement-based floors in some households, and leave other households with their current floors and then compare disease rates in these two groups of households. This study will take place in villages in two counties (one in Kwale and one in Bungoma) and will last for about one year. In order to make it fair, at the end of the study the households that were originally left without a new cement-based floor will be provided with the same new cement-based floor that other households received.

Why have I been asked to participate?

You have been invited to participate in this study because you live in the community where this study is taking place, there is a child under the age of five who lives in your household, you currently do not have an improved floor in your home, and your home has been deemed to be eligible to have a floor installed without risking structural damage.

Do I have to participate? Can I change my mind about taking part?

You are free to decide whether participate or not. If you don't want to take part, that's ok. You are also free to stop participating in this study at any time for any reason. If you withdraw from the study, we will keep any information you have provided to us confidential and exclude it from our analyses and study reports.

What will I have to do and what will happen to me if I take part?

<u>The floor</u>

If you agree to participate in this trial your household will be randomly assigned to have a new cement-based floor installed in your home **either** now (within the next 1-4 months) **or** after one year (at the end of the study). The floor will be installed in all buildings occupied by your immediate household with the exception of animal shelters and latrines. **Installation will take up to seven days** and will require all of your belongings to be temporarily removed and for your household to relocate for this period. You may also be invited to join a small group of neighbouring participating households in meeting every few months to discuss the floor.

<u>The research</u>

You will be visited by members of the study team at different points over the next 15 months who will conduct different survey activities at your home. These will include:

- A household questionnaire that will be administered once now, and once in 12 months and will collect information on who lives at your household, what belongings and animals you own, the GPS location of your household, and water and sanitation arrangements. The questionnaire will take around 30 minutes.
- Collecting soil on your floor from different places in your home; including the room where your child sleeps, the area where you cook and prepare food, the courtyard of your home, and the entrance to your room used most (busy room). This will be to see if parasites and other microorganisms can be found on the floor. This will be done once in about 6 months and again after about 12 months.
- Regular unscheduled visits by field officers to inspect the condition of the floor and how it is being used. Photos of the floor and wider dwelling may be taken but only with the verbal consent of present adults.

- In addition to this we may ask members of your household to participate in some additional research activities that could include; providing stool samples, undergoing assessments for presence of jigger fleas, questionnaires about quality of life; and observations of daily routines. We will provide further information about these activities as we come to them and will ask you again for your informed consent before undertaking them.

What are the possible risks and disadvantages? Trained fundis will oversee the installation of the floor in your home and the utmost care will be taken to ensure that it is completed to a high standard, however it is possible that installing the floor may cause some superficial or structural damage to the existing building. Participating in group meetings will take up some of your time and will require someone from your household to travel to a central location within the village. The household questionnaire and other data collection activities will take up some of your time.

What if something goes wrong? If you are unhappy with any aspect of this study, you should speak to the researchers who will do their best to address your concerns. If you remain unhappy and wish to complain formally you can contact Patricia Henley using the contact details listed at the bottom of this sheet.

What are the possible benefits? You will receive a new cement-based floor in your home and will be offered treatment for parasitic worm infections. In addition to this, the information you provide will help us to understand if improved floors are linked to better health and wellbeing. This could be used by governments and other organisations to improve the health and wellbeing of communities like yours in Kenya and further abroad.

What will happen to information collected about me?
All information collected about you will be kept private and it will not be shared with anyone else. Only a small number of researchers within the study team will be allowed to look at information about you such as your name, contact details and village of residence. At the end of the project, your name will be removed from study data and remaining data will be kept in an online data store. The data will be made available to other researchers worldwide for research and to improve medical knowledge and patient care. However, your personal information will not be included and there is no way that you can be identified. Photos taken of your home will be stored electronically for five years before being deleted. These photos may be published as part of reports or academic papers which means they could be viewed by anyone anywhere in the world. Published photos may remain publicly available for a period of over five years.

What will happen to the results of this study? The study results will be shared with the government and organisations within Kenya and published in scientific journals so that other researchers can learn from them. Your personal information will not be included in the study reports. However, photos of your home may be included in these reports.

Who has reviewed this study? All research involving human participants is looked at by an independent group of people, called a Research Ethics Committee, to protect your interests. This study has been reviewed and approved by The London School of Hygiene & Tropical 's Research Ethics Committee and the Kenya Medical Research Institute's Scientific Ethics Review Unit.

Further information and contact details Thank you for taking time to read this information sheet. If you would like to participate in the study, please read and sign the consent form. If you have any questions or concerns, you can ask the researchers who will do their best to answer your questions. You may also contact any of the Study Investigators listed below[1]:

Dr. Charles Mwandawiro
Kenya Medical Research Institute
P.O Box 54840-00200, Nairobi / Telephone : 020 272 2541. Mobile : 0772476836
Dr. Rachel Pullan
London School of Hygiene and Tropical Medicine
London WC1E 7HT, UK / Telephone : +44 (0)20 7927 2702
Dr. Ulrike Fillinger
International Centre for Insect Physiology and Ecology (ICIPE)
P.O. Box 30, 40305 Mbita, Mbita District, Kenya / Tel: +254-59-22216/7/8

If you want to ask someone independent anything about this research, please contact:

KEMRI	LSHTM
The Secretary<br>KEMRI Scientific and Ethics Review Committee<br>P.O Box 54840-00200, Nairobi<br>Tel: 020 272 2541 Mobile 0722205901 mobile; 0717 719477 / Email: seru@kemri.org	Patricia Henley<br>Head of Research Governance and Integrity<br>London School of Hygiene and Tropical Medicine<br>London WCE1 7HT, UK<br>Tel: +44 (0) 20 7927 2626 / Email: rgio@lshtm.ac.uk

[1] Contact details of the research focal person in the study site will provided as soon as he/she is recruited.

Consent form 201a: household study enrolment

Understanding the contribution of household flooring to disease burden in rural Kenya

Name of PI/researcher responsible for project: Dr Charles Mwandawiro / Dr Rachel Pullan / Dr Ulrike Fillinger

I, ______________________________ confirm that I have read the information sheet for the above-named study OR I have had the information explained to by study personnel in a language that I understand. I have had the opportunity to consider the information, ask questions and have these answered satisfactorily. I understand that my participation is voluntary and that I am free to withdraw at any time without giving any reason, without my legal rights being affected.

Please circle all you agree with		Head of household to complete
Yes	No	I agree to allow study-trained fundis to install a cement-based floor within my home
Yes	No	I agree to allow photos that are taken of my home to be published within reports and academic papers and understand that this will make them publically accessible
Yes	No	I agree to take part in the above study and give permission to the researchers in this study to conduct data collection activities as described in this consent form
Yes	No	I understand that data about/from me may be shared with other researchers to support other research in the future, and that I will not be identifiable from this information.

**Printed name of participant**	**Signature (or thumbprint*) of participant**	**Date**
		|__|__|/|__|__|/|__|__|

Only necessary if the participant cannot read:
I *attest that the information concerning this research was accurately explained to and apparently understood by the participant and that informed consent was freely given by the subject/parent/guardian.

**Printed name of impartial witness***	**Signature of impartial witness***	**Date***
		|__|__|/|__|__|/|__|__|

I attest that I have explained the study information accurately in ________________________ and was understood to the best of my knowledge by the participant and that he/she has freely given their consent to participate in the presence of the above named impartial witness (where applicable)

**Printed name of person obtaining consent**	**Signature of person obtaining consent**	**Date**
		|__|__|/|__|__|/|__|__|

[*Only required if the participant is unable to read or write.]

This signed informed consent document to be kept by the participant
