# Supplementary material for: Evaluating impacts of improved flooring on enteric and parasitic infections in rural households in Kenya: study protocol for a cluster-randomised controlled trial
Source: BMJ Open. 2025 Jun 6;15(6):e090464. doi: 10.1136/bmjopen-2024-090464 (PMC12161379; doi:10.1136/bmjopen-2024-090464)
Supplement: Supplementary file 3 [file bmjopen-2024-090464.reviewer_comments.pdf]

PEER REVIEW HISTORY

BMJ Open publishes all reviews undertaken for accepted manuscripts. Reviewers are asked to complete a checklist review form and are provided with free text boxes to elaborate on their assessment. These free text comments are reproduced below.

ARTICLE DETAILS

Title (Provisional)

Evaluating impacts of improved flooring on enteric and parasitic infections in rural households in Kenya: Study protocol for a cluster-randomised controlled trial

Authors

Halliday, Katherine E; Kepha, Stella; Legge, Hugo; Allen, Elizabeth; Dreibelbis , Robert; Elson, Lynne; Kakoi, Beatrice K; Mcharo, Carlos; Muli, Sharon; Mwongeli, Jacinta; Njomo, Doris; Njoroge, Margaret M; Ochwal, Victoria; Oswald, William E; Rono, Martin; Safari, Tuva K; Filinger, Ulrike; Kaluli, James Wambua; Mwandawiro, Charles S; Pullan, Rachel

---

VERSION 1 - REVIEW

**Reviewer**	**1**
**Name**	**Zirimenya , Ludoviko**
**Affiliation**	**MRC/UVRI and LSHTM Uganda Research Unit, Immunomodulation and Vaccines Programme**
**Date**	**04-Aug-2024**
**COI**	**None**

Stakeholder Engagement and Formative Work: The manuscript mentions that the intervention and assessment methods were informed by extensive stakeholder engagement from local to national levels, as well as in-depth formative work. If this formative work has been published, it would be helpful to provide a reference in the methods section. This would allow readers to understand how these factors informed the intervention and assessment methods. Specifically:

- What is the rationale for choosing cement-based flooring as an intervention to address the target diseases?
- Were alternative interventions considered, and if so, why was cement-based flooring ultimately selected?

Rationale and Mechanisms:

- Is there a clear explanation of how cement-based flooring is expected to reduce the burden of the target diseases? What are the underlying mechanisms?
- The Theory of Change is mentioned, but as this resource is still in press and not accessible, it would be beneficial if the authors provided a clear explanation of how the cement-based flooring is expected to achieve its intended outcomes and the rationale behind its selection.

Implementation Consistency:

- How will the intervention be implemented consistently across clusters?
- Will the same locally-recruited masons be used for constructing the floors in all 220 households? If not, what measures are in place to ensure consistency in implementation across households?

Sustainability Evaluation:

- What are the plans to evaluate the sustainability of the intervention beyond the study period?

Addressing Loss to Follow-Up:

It is noted that households intending to move within the next 12 months will be excluded. However, it is possible that enrolled households may move for unexpected reasons. How will the study address potential loss to follow-up due to such relocations?

Reviewer 2

Name Hürlimann, Eveline

Affiliation Schweizerisches Tropen- und Public Health-Institut, Medical Parasitology and Infection Biology

Date 11-Oct-2024

COI I don't have any competing interests.

General comments:

The authors present the protocol for a cluster-randomized trial to evaluate the effect of improved flooring on enteric and parasitic infection prevalence and intensity in two different sites in Kenya. Cement or tile-flooring of housing or sanitation facilities has partly been shown to be related to reduced infection burden, however, the current evidence for its potential direct effect is scarce. The research question is certainly of interest to be explored. In view that the intervention could be considered a WASH intervention I would recommend the authors to be very clear in the definition of primary outcomes, interventions that have an effect on the targeted infections/parasites (including MDA with anthelminthics/antiparasitic treatments), starting point for all households to be evaluated (do you treat them all first? how to take into account baseline infection rates?), assessment time points (WASH interventions need time to produce measurable effect) otherwise they risk to not find any effects. Some of these points become clearer once going through the manuscript and from looking at the figures/supplement, please make sure it is already clear also from looking at the abstract. The quite low expected baseline prevalence of STH can pose a problem to find any measurable effect, worst case you may need to adapt your primary outcome. I also don’t understand why no floor samples are taken at baseline if environmental contamination is suspected to be one of the main drivers for infection in the settings. It would make sense to check for it before installing any new floors (at least in a subsample of the households) and not only check at 12 months past intervention start.

The manuscript is well written and does not need further language editing. Some sections may profit from some more detailed description (e.g. lab work, medical interventions/treatments, why certain assessments are done and how evaluated exactly, the

questionnaire and self-reported illness part is a bit vague and risks to produce less valuable data). The process evaluation seems a little bit off from a trial protocol description, yet important to understand how the intervention worked out, maybe rename it?

Please add a SPIRIT checklist as commonly done for protocol manuscripts.

Specific comments:

Abstract:

- I would better explain methodology (interventions, assessment time points, evaluations) and skip the ethics/dissemination part in the abstract. From the abstract it is not clear that you treat the household members after the baseline assessment for example. Environmental contamination analysis is left out although very relevant in view of the type of intervention.

Aims and objectives: please be more specific what you mean by “reducing the burden”, burden in terms of what? (prevalence reduction? intensity reductions?)

Methods and analysis:

- Study design summary: How many stool samples do you collect per assessment time point? please state tungiasis treatment scheme or provide proper reference. Why don’t you do an environmental survey at baseline (how do you want to measure a reduction in contamination if you only do it once?) ? What is the situation in the study setting with regard to enteric infections/diarrheal disease burden in young children? In my view “to provide improved flooring to control households at study end” is not part of the design but rather an ethical consideration and should be moved there (I see it is already there so can be removed here). I would also not include it in Figure 1.

- Intervention: it is a pity the improved floor is not applicable to unimproved pit-latrines, I would expect quite some benefit from improving the slab of these kind of latrines. How high is the proportion of these kind of latrines in the planned study sites? Wouldn’t you expect then a decreased effect if you just improve the house but not the toilet?

- Trial outcomes: 1) ideally you have ONE single primary outcome that you then also use for randomization/allocation of households to either control or intervention to have comparable groups from start. I would choose the most common outcome among the three to then randomize (in your case probably the one you assess in all household members? STH infections?). 2) environmental contamination analysis should be clearly stated as a secondary outcome (also in the abstract). 3) How often do you plan to ask caregivers for gastrointestinal illness (diarrhea) of their younger children? This information is only useful if this is done on a weekly or if not feasible on a two-weekly basis in view of the nature of diarrhea episodes. If you just ask at baseline and then 12 months after only you will not get any valuable data and can directly skip it. 4) It is not entirely clear to me for what you are doing the QoL questionnaires with children and adults. You should have a clear idea what you want to draw out of these (do you do a scoring and compare before and after?). Please keep in mind that this is a lot of work to do and creates a lot of extra data that is in the end of such projects often not even touched because given less priority and often not clear what to take out of it…

-Sample size calculation: 15% loss to follow-up seems unrealistic for a community-based study over a period of 12 months (15% is often used for short-termed pre-post assessments such as drug efficacy studies). In this kind of trials I would rather expect a loss to follow-up between 30-50%. If you recruit try to opt for more since you also have a number of different outcomes you assess and there will always be participants with missing assessments.

- Randomisation: Randomisation should be based on your primary outcome (STH baseline infection status) in order to have comparable groups per site. Even if you have an established way to make them draw the allocation sign, you may still want to arrange them by infection status at household level (households with STH infection vs no infections; you could arrange it as if you would do a stratified randomization).

- Assessments: Same comment as above, I don't understand why you only do an environmental survey at 12 months but not at baseline. Also why you leave out sanitation facilities for sampling? Why not check for STH as well when you do PCR anyway (Kato-Katz examination of one single stool sample is potentially underestimating the true burden, is it too costly?)?

-Laboratory work: very scarcely written, no standard references for the different techniques provided (except for entomology sweeps), no quality assurance/control mechanisms for the different techniques stated.

-Statistical analysis: What do you mean by appropriate effects? Please precise what are appropriate effect measures for your primary outcome(s). Wouldn't you rather include covariates such as socioeconomic variables into adjusted regression models rather than using interaction terms?

Ethics and dissemination:

-Please provide reference numbers for ethical approvals.

-Please describe consenting procedures for illiterate participants.

VERSION 1 - AUTHOR RESPONSE

Reviewer 1 Dr. Ludoviko Zirimenya, MRC/UVRI and LSHTM Uganda Research Unit

Stakeholder Engagement and Formative Work:

1. The manuscript mentions that the intervention and assessment methods were informed by extensive stakeholder engagement from local to national levels and in-depth formative work. If this formative work has been published, it would be helpful to provide a reference in the methods section. This would allow readers to understand how these factors informed the intervention and assessment methods

The paper describing the formative work has been published (Legge H, Kazungo K, Muli S, Elson L, Mwongeli J, Halliday KE, et al. Identifying Potential Determinants of Faecal Contamination on Domestic Floors in Three Settings in Rural Kenya: A Mixed Methods Analysis. Environ Health Insights. 2024;18:11786302241246454. doi: 10.1177/11786302241246454) and is cited in line 100 (of the marked copy) at the end of the introduction. We have also included the citation at other points in the methods section where the formative work is referred to.

"The Intervention design was guided by mixed methods formative research [1]."

"Initially, formative work was carried out in 2021 in Kwale, Bungoma, and Narok Counties to: identify housing

conditions, map daily routines, and explore home improvement priorities to inform intervention development [1].”

“Formative research indicated potential for heterogeneity in how household members interact with the floor and adapt behaviours such as cooking, animal husbandry, and sleeping, all of which may play an important role in mitigating intervention success [1].”

The stakeholder engagement is covered in the Theory of Change (TOC) paper, which has now been accepted by BMC Public Health (DOI:10.1186/s12889-025-21469-1) and we expect it to be published imminently at which point we can fully update the citation. We have added a reference to this manuscript in the “Sensitisation and engagement” section (lines 277-279) .

“Following formative research, consultative meetings were held at the ward- and village-level, presenting research findings and compiling input on acceptability and ownership of the proposed intervention [2].”

2. What is the rationale for choosing cement-based flooring as an intervention to address the target diseases? Were alternative interventions considered, and if so, why was cement-based flooring ultimately selected?

The rationale for choosing cement-stabilised flooring is based on the need to develop a sealed, washable floor which is durable, and smooth, with acceptable appearance, good wear resistance, and is affordable. Alternative interventions were considered, including off-products of production such as bagasse from sugar cane production. However, these products were either already being used or not available in large- enough quantities to procure locally. The decision was also informed by a recent feasibility study conducted by members of the study team who explored locally appropriate, low-cost flooring solutions to create a sealed, washable floor for the prevention of tungiasis [3]. Floor slabs were tested using a range of materials to bind local-site subsoil including fire ash, coconut fibres, a mix of lime, sodium hydroxide and silicate with or without cement. The slab with subsoil mixed with cement at a 1:9 ratio was among the best performing with the lowest material cost per m^2. The decision to use cement-stabilised floors earthen

floors built on these findings and implemented improvements such as adding a damp-proof membrane. A separate manuscript is planned to describe the floor selection and floor properties in more detail. However, here we have now included some text and the reference to this feasibility study to provide the reader with more information on the rationale behind the selection of cement-based flooring (lines 181- 186).

"A low-cost, cement-stabilised earthen floor is installed in each eligible building of the dwelling (excluding unimproved pit-latrines, dedicated animal sheds, and stores) to meet the following requirements: (i) non-absorbent, durable, and smooth; (ii) good wear resistance; (iii) acceptable appearance; and (iv) affordable. The decisions on flooring material and construction were informed by and built on a recent feasibility study exploring locally appropriate, and affordable flooring solutions to create a sealed, washable floor for the prevention of tungiasis in coastal Kenya [3]. "

Rationale and Mechanisms:

3. Is there a clear explanation of how cement-based flooring is expected to reduce the burden of the target diseases? What are the underlying mechanisms? The Theory of Change is mentioned, but as this resource is still in press and inaccessible, it would be beneficial if the authors provided a clear explanation of how the cement-based flooring is expected to achieve its intended outcomes and the rationale behind its selection

The TOC manuscript describes the TOC and intervention development and how the workshops and stakeholder meetings shaped these. This manuscript has been accepted by BMC Public Health (DOI: 10.1186/s12889-025-21469-1) and we expect it to be published online imminently, at which point we will fully update the citation. However, for now we have also expanded on the overview of how cement-based flooring is expected to achieve its intended outcomes in this manuscript (lines 166-178).

"Briefly, installation and ongoing maintenance of improved household flooring is expected to reduce transmission of enteric and parasitic infections, by preventing direct exposure to pathogen and parasite contamination and through an intermediate effect of improved domestic hygiene. This is predicated on the assumptions that household members spend their time on the improved floor; it is a sealed surface limiting the opportunity for STH and sand flea off-host stages; it is washable and is regularly cleaned to reduce pathogen build-up and has limited animal contact to minimise faecal contamination. Improved household flooring is also intended to improve caregiver wellbeing through a number of routes including reduced health anxiety [4], improved comfort [5], and increased levels of satisfaction, pride and self-efficacy [6 7]. Child wellbeing could be impacted through increased energy levels resulting from decreased exposure to enteric pathogens, and reduced pain and itching as well as improved mobility, sleep patterns and concentration resulting from reduced exposure to skin parasites such as Tunga penetrans [8-10]."

Implementation Consistency:

4. How will the intervention be implemented consistently across clusters? Will the same locally recruited masons be used to construct the floors in all 220 households? If not, what measures are in place to ensure consistency in implementation across households?

We agree this is an important consideration. The masons are recruited locally at each site, so one group of masons install the floors in Kwale and another set of locally recruited masons install the floors in Bungoma. To ensure consistency in implementation across households and sites: (1) masons at both sites are trained by the same Master Trainers; (2) the field supervisors supervising the process are the same across sites; (3) a set of locally-recruited field officers oversee the masons with a standardised checklist to ensure consistency; and (4) the masons from Bungoma travelled to Kwale to observe the Kwale masons at work, and the Kwale masons travelled to Bungoma to partake in the training and offer advice based on lessons learnt. We have expanded on this within the manuscript text (lines 194-199)

"All materials are provided by the study. Installation is performed by locally-recruited masons, trained by the study team, with the support of local laborers. The study team provides day-to-day supervision and quality checks using a standardized checklist for all flooring activities, and the process is overseen by the same supervisors across sites. Additionally, to ensure consistency across sites, the masons from Bungoma travel to Kwale to observe the masons at work, and the Kwale masons travel to Bungoma to partake in the training and offer advice based on lessons learnt"

5. **Sustainability Evaluation:** What are the plans to evaluate the sustainability of the intervention beyond the study period?

We agree that evaluating the sustainability of the improved floors beyond the initial 12-month evaluation period would be extremely valuable. Our current funding restricts the study follow-up period to 12 months. Therefore, it is listed as a limitation in the "Strengths and Limitations of Study" Section (line 64- 65):

"Study follow-up is limited to 12 months, and so the study will not be able to evaluate longer term durability, sustainability and impact of the flooring intervention."

6. **Addressing Loss to Follow-Up:** It is noted that households intending to move within the next 12 months will be excluded. However, enrolled households may move for unexpected reasons. How will the study address the potential loss of follow-up due to such relocations?

As mentioned, we have tried to anticipate this as much as possible, by enrolling families who report no plans to move in the next 12 months. However, we realise this is not always known at the time. If enrolled families move house, but remain with the study sites, we follow up the families in their new location and note that they have moved, when they moved and their current living situation. We have added text at the start of the "Assessments" section to explain this (lines 310-312).

"The assessments undertaken are described below and outlined in Online supplemental material. Once enrolled, any household which moves house is followed up in their new location, with a note made of this, providing they are still within the study sites."

As stated in the "Statistical analyses" section, the primary analyses will be conducted using intention-to- treat so will include these families followed up in their new location. However, we appreciate that families may relocate out of the study area, and it is beyond the scope of the study to follow them so these will be lost to follow-up (lines 396-397).

"Analysis of primary and secondary human infection outcomes will be carried out on groups as randomised (intention-to-treat)."

Reviewer: 2 Dr. Eveline Hürlimann, Schweizerisches Tropen- und Public Health-Institut

The authors present the protocol for a cluster-randomized trial to evaluate the effect of improved flooring on enteric and parasitic infection prevalence and intensity in two different sites in Kenya. Cement or tile-flooring of housing or sanitation facilities has partly been shown to be related to reduced infection burden, however, the current evidence for its potential direct effect is scarce. The research question is certainly of interest to be explored.

In view that the intervention could be considered a WASH intervention I would recommend the authors to be very clear in the definition of primary outcomes, interventions that have an effect on the targeted infections/parasites (including MDA with anthelminthics/antiparasitic treatments), starting point for all households to be evaluated (do you treat them all first? how to take into account baseline infection rates?), assessment time points (WASH interventions need time to produce measurable effect) otherwise they risk to not find any effects. Some of these points become clearer once going through the manuscript and from looking at the figures/supplement, please make sure it is already clear also from looking at the abstract.

Thank you, in addressing your comments below we hope we have now clarified any uncertainty around the definitions of the primary outcomes and interventions and any treatment provided following health assessments performed. Unfortunately, a longer intervention period is beyond the scope of the study, and so is mentioned as a limitation upfront.

The quite low expected baseline prevalence of STH can pose a problem to find any measurable effect, worst case you may need to adapt your primary outcome. I also don't understand why no floor samples are taken at baseline if environmental contamination is suspected to be one of the main drivers for infection in the settings. It would make sense to check for it before installing any new floors (at least in a subsample of the households) and not only check at 12 months past intervention start.

We address the collection of environmental samples at post-intervention only in responses to comments below.

The manuscript is well written and does not need further language editing. Some sections may profit from some more detailed description (e.g. lab work, medical interventions/treatments, why certain assessments are done and how evaluated exactly, the questionnaire and self-reported illness part is a bit vague and risks to produce less valuable data). The process evaluation seems a little bit off from a trial protocol description, yet important to understand how the intervention worked out, maybe rename it?

1. Please add a SPIRIT checklist as commonly done for protocol manuscripts.

We have completed and uploaded the SPIRIT checklist

Abstract:

2. I would better explain methodology (interventions, assessment time points, evaluations) and skip the ethics/dissemination part in the abstract. From the abstract it is not clear that you treat the household members after the baseline assessment for example. Environmental contamination analysis is left out although very relevant in view of the type of intervention.

We agree these are important points to include in the abstract. However, we are restricted by the abstract word limit of 300 words and the prescribed abstract structure for this article type so we are unable to remove the ethics/dissemination section. To try and address your comment we have now added a sentence to indicate individuals were treated following baseline assessments (lines 35-39). However, we have had to cut words in other areas to facilitate this.

*"**Methods and analyses**: 440 clusters (households) across both sites are allocated to control or intervention group, in which a low-cost, sealed, washable, cement-based floor is installed in eligible*

buildings of the dwelling, alongside a floor care guide provided during an induction meeting. Following baseline assessments in both groups, all individuals over 1 year receive albendazole and those infected with tungiasis receive benzyl benzoate."

Environmental contamination is already listed under the secondary outcomes, so we have left that as is (lines 42-46).

"Secondary outcomes include: prevalence of caregiver-reported gastrointestinal illness in children under 5 years; intensity of STH and tungiasis infections; quality of life and wellbeing measures; and environmental contamination."

Aims and objectives:

3. Please be more specific what you mean by "reducing the burden", burden in terms of what? (prevalence reduction? intensity reductions?)

In describing the overall aim we use the term burden to cover both our primary and secondary outcomes. Our primary outcomes are prevalence reductions of enteric infections, STH and tungiasis. Secondary outcomes include reductions in intensity. In order to clarify this, we have now modified this section to explicitly list intensity of STH infections and tungiasis under the secondary objectives (lines 102-108), similarly to how it is listed under the "Trial outcomes" section later on in the manuscript.

"Our primary aim is to evaluate the effectiveness of an improved (cement-based) flooring intervention in reducing the burden of enteric infections, STH and tungiasis in participating households…"

"The primary objectives are to quantify impact on the prevalence of enteric infections, STH infections, and tungiasis. Secondary objectives include assessing impact on the intensity of STH infections, and tungiasis, wellbeing of caregivers and children, self-report gastrointestinal illness in children, and the extent to which entero-pathogen and parasitic contamination of floors is reduced within the home."

Methods and analysis: Study design summary:

4. How many stool samples do you collect per assessment time point?

We collect one stool sample per individual per time point. We realise that duplicate samples increase the reliability of the Kato-Katz method, but this was not logistically feasible in this case. To clarify this, we have added that a single stool sample per individual is collected (lines 325-327):

"*During the **stool survey** all household members are invited to provide a single stool sample at 0 and 12 months for screening for enteric infections (children under five years) and STH infections (all residents over one year)*."

5. Please state tungiasis treatment scheme or provide proper reference.

We have now clarified the tungiasis treatment scheme provided as below in both the "Study design summary" (lines 129-131) and "Ethics and dissemination" (lines 437-439) sections, and have provided references to the National Tungiasis Prevalence Survey and a study characterising tungiasis infection and morbidity during COVID-19 school closures, which both used the same treatment in consultation with County Health Managers. We have also included this information in the abstract, as indicated in response to Comment 2 above.

"During tungiasis assessments, those found to be affected are treated with benzyl benzoate (BB), provided by the study, at the point at which they are found, and the BB lotion is provided to the relevant community health volunteer to follow up with two further treatments [11 12]."

"All tungiasis cases detected at baseline and endline are treated with BB lotion, provided by the study, and the BB lotion is provided to the relevant community health volunteer to follow up with two further treatments [11 12]*."*

6. Why don't you do an environmental survey at baseline (how do you want to measure a reduction in contamination if you only do it once?)

The analysis will be based on the difference between control and intervention groups at endline rather than a before and after analysis. We are assuming true randomisation and so there should be no reason to expect a systematic difference between groups at baseline. This is a secondary outcome, and if the primary outcomes are balanced between arms it's a reasonable assumption that floor contamination will also be balanced. Also, the formative work indicates that the houses are very similar across multiple characteristics in the study area.

7. What is the situation in the study setting with regard to enteric infections/diarrheal disease burden in young children?

We have now added the prevalence of diarrhea in children under 5, according to the latest DHS survey (2022) in the "Study settings" section (lines 154-156 and lines 159-161).

"Within Kwale…STH prevalence (predominantly hookworm) was 20% in 2017 [13]*, reported diarrhea prevalence in children under 5 was 3.2% in 2022* [14 15]*,and tungiasis prevalence has been found to be greater than 50% in some villages* [16]*."*

"Within Bungoma…Prevalence of any STH in school-age children is 7.3% [17]*, reported diarrhea prevalence in children under 5 was 18% in 2022* [14 15] *and tungiasis has been reported as present."*

8. In my view "to provide improved flooring to control households at study end" is not part of the design but rather an ethical consideration and should be moved there (I see it is already there so can be removed here). I would also not include it in Figure 1.

We have removed mention of this from the Study Design Summary section of the manuscript (lines 139- 140), and have also moved the mention of this in the abstract from the Methods and Analyses section to the Ethics and Dissemination section (lines 50-51). We have also removed the box referring to this from Figure 1.

Intervention:

9. It is a pity the improved floor is not applicable to unimproved pit-latrines, I would expect quite some benefit from improving the slab of these kind of latrines. How high is the proportion of these kind of latrines in the planned study sites? Wouldn't you expect then a decreased effect if you just improve the house but not the toilet?

There has been a reasonable amount of research examining the effect of improved latrines, whereas this is not the case for improved flooring. Here we are interested to know if improving domestic floors has an effect on these pathogens and parasites regardless of latrine status. During the formative work, we found 15% of households in Kwale and 17% in Bungoma had unimproved latrines (latrines without a slab). We have now included this information in the "Study settings" section (lines 158-163) . If we were to improve latrines, we would need to do this across both study groups, or change to a factorial design to look at the effects of improving latrines and flooring independently. Therefore, we have excluded latrine buildings from the list of eligible buildings so as not to implement an improved sanitation intervention and improved flooring intervention simultaneously.

"Eighty-percent of house floors were earthen and 15% had unimproved (without slab) latrines. Within Bungoma County the study villages span South Bukusu and Kabula wards in Bumula Sub-County. Prevalence of any STH in school-age children is 7.3% [1]*, reported diarrhea prevalence in children under 5 was 18% in 2022* [14 15] *and tungiasis has been reported as present. Formative work among the seven*

study villages censused 4560 individuals living across 906 households, the majority of which (77%) had earthen floors and 17% of which had unimproved latrines [1].”

Trial outcomes:

10. Ideally you have ONE single primary outcome that you then also use for randomization/allocation of households to either control or intervention to have comparable groups from start. I would choose the most common outcome among the three to then randomize (in your case probably the one you assess in all household members? STH infections?).

We are assuming true randomisation leading to sufficient comparability between study groups. In the trial results paper, we will present a table of the baseline characteristics of the households by study arm to confirm true randomisation and that there is comparability across characteristics at baseline, but we have no reason to assume they will not be comparable and no significance testing of baseline differences will be performed. The baseline data on the primary outcomes is blinded and locked and will only be looked at by the unblinded trial statistician during the final analysis.

11. Environmental contamination analysis should be clearly stated as a secondary outcome (also in the abstract).

Thank you, we have now modified the text to clarify that the environmental contamination analyses are secondary outcomes (lines 224-227).

“To quantify reduction in contamination, secondary outcomes measured in both arms through environmental surveys of soil/dust samples include: contamination of floors with the same pre- specified enteric pathogens and STH eggs/larvae (measured by PCR) and with eggs, larvae, pupae, and adults of T. penetrans (measured using entomologic screening).”

Environmental contamination is listed under the secondary outcomes in the abstract, so we have left that as is (lines 42-45).

“Secondary outcomes include: prevalence of caregiver-reported gastrointestinal illness in children under 5 years; intensity of STH and tungiasis infections; quality of life and wellbeing measures; and environmental contamination.”

12. How often do you plan to ask caregivers for gastrointestinal illness (diarrhea) of their younger children? This information is only useful if this is done on a weekly or if not feasible on a two-weekly basis in view of the nature of diarrhea episodes. If you just ask at baseline and then 12 months after only you will not get any valuable data and can directly skip it.

This question is only asked at baseline and 12 months, and thus is prevalence of reported gastrointestinal illness, rather than incidence. We appreciate that the usefulness of this question at a single timepoint is limited, and when it is presented as a secondary outcome, it shall be presented in the context of its limitations.

13. It is not entirely clear to me for what you are doing the QoL questionnaires with children and adults. You should have a clear idea what you want to draw out of these (do you do a scoring and compare before and after?). Please keep in mind that this is a lot of work to do and creates a lot of extra data that is in the end of such projects often not even touched because given less priority and often not clear what to take out of it…

The WHO-5, WHOQOL-bref, and EQ5DY are validated tools with pre-specified methods for aggregating wellbeing and quality-of-life scores. Analysis will be between study arms at endline with baseline measurements presented to demonstrate comparability of scores at prior to the intervention. The

rationale behind this outcome is explored in the TOC manuscript which has been accepted by BMC Public Health (10.1186/s12889-025-21469-1) and we expect it to be published imminently. Improved household flooring could improve caregiver wellbeing through a number of routes including reduced health anxiety [4], improved comfort [5], and increased levels of satisfaction, pride and self-efficacy [6 7]. Child wellbeing could be impacted through increased energy levels resulting from decreased exposure to enteric pathogens and improved sleep patterns resulting from reduced exposure to skin parasites such as *Tunga penetrans* [8-10]. We have added text into the manuscript to explain these possible pathways (lines 173-178). This secondary outcome will be presented in the trial results paper and further exploratory analyses are planned bringing in the observation and interview data to supplement the QoL questionnaire findings.

"*Improved household flooring is also intended to improve caregiver wellbeing through a number of routes including reduced health anxiety [4], improved comfort [5], and increased levels of satisfaction, pride and self-efficacy [6 7]. Child wellbeing could be impacted through increased energy levels resulting from decreased exposure to enteric pathogens and reduced pain and itching as well as improved mobility, sleep patterns and concentration resulting from reduced exposure to skin parasites such as Tunga penetrans [8-10].*"

14. **Sample size calculation:** 15% loss to follow-up seems unrealistic for a community-based study over a period of 12 months (15% is often used for short-termed pre-post assessments such as drug efficacy studies). In this kind of trials I would rather expect a loss to follow-up between 30-50%. If you recruit try to opt for more since you also have a number of different outcomes you assess and there will always be participants with missing assessments.

We are very familiar with the study settings we are working in and our estimate of loss to follow-up is based on our experiences and on previous community-based interventions. In a Cochrane review of "Interventions to improve water quality and supply, sanitation and hygiene practices, and their effects on the nutritional status of children (Review)" in the seven studies that reported attrition, it ranged from 4% to 16.5%" [18].

Please also see our response to Comment 6 of Reviewer 1 regarding loss to follow-up.

15. **Randomisation:** Randomisation should be based on your primary outcome (STH baseline infection status) in order to have comparable groups per site. Even if you have an established way to make them draw the allocation sign, you may still want to arrange them by infection status at household level (households with STH infection vs no infections; you could arrange it as if you would do a stratified randomization).

Please see our response to Comment 10 above. We are assuming true randomisation resulting in sufficient comparability between study groups. The randomisation was stratified by village within study site.

Assessments:

16. Same comment as above, I don't understand why you only do an environmental survey at 12 months but not at baseline.

Please see our response to Comment 6 above

17. Also why you leave out sanitation facilities for sampling?

Please see our response to Comment 9 above.

18. Why not check for STH as well when you do PCR anyway (Kato-Katz examination of one single stool sample is potentially underestimating the true burden, is it too costly?)?

The primary STH outcome in all individuals >1 year is assessed via Kato-Katz. We are only conducting PCR on stool samples collected from under 5s to assess enteric infection. As we are not running PCR on

individuals over 5 years, we need to ensure the same diagnostic across all age groups for this primary outcome. Whilst we acknowledge KK has lower sensitivity, PCR on all stool samples would be prohibitively costly.

19. **Laboratory work:** very scarcely written, no standard references for the different techniques provided (except for entomology sweeps), no quality assurance/control mechanisms for the different techniques stated.

We have now added information on the QA/QC mechanisms and added references to the sections related to the Kato-Katz and PCR (lines 363-367, 368-371, 376-379)

"Laboratory work

Stool samples from children <5 years are aliquoted into cryovials for storage in 95% ethanol at -80°C. DNA is extracted from the stool using the QIAGEN® QIAamp® DNA Mini Kit [19] and real-time quantitative PCR is used for the detection of pre-specified enteric pathogens: Giardia lamblia, Cryptosporidium spp., Campylobacter jejuni, enteroaggregative E. coli (EAEC), Shigella and adenovirus.

Stool samples from individuals >1 year are processed and examined in duplicate (41.7mg template) for presence and quantity of STH eggs using the Kato-Katz method [20]. Duplicate slides are prepared from the single day stool samples and are read by independent microscopists with a 10% quality control check performed.

DNA is extracted from soil samples collected during the environmental surveys according to specifications outlined in the QIAGEN® DNeasy® PowerSoil® Pro documentation (MO BIO Laboratories, Inc., Carlsbad, CA) [21] and is assessed for the pre-selected enteric pathogens and STH egg/larvae DNA using the established real-time PCR panel."

Statistical analysis:

20. What do you mean by appropriate effects?

Apologies, this should have said as appropriate effect sizes. We have now amended this in the text below (lines 397-399).

"Results will be presented as appropriate effect sizes (risk ratios for the primary outcomes) with a measure of precision (95% CIs), using generalised estimating equations with robust standard errors to account for correlation of outcomes within households."

21. Please precise what are appropriate effect measures for your primary outcome(s).

The effect measures for the primary outcomes (prevalence of enteric infections, STH and tungiasis) will be risk ratios with generalised estimating equations used to estimate the difference between study groups after 12 months of intervention. We have added this into the text (lines 397-399).

"Results will be presented as appropriate effect sizes (risk ratios for the primary outcomes) with a measure of precision (95% CIs), using generalised estimating equations with robust standard errors to account for correlation of outcomes within households."

22. Wouldn't you rather include covariates such as socioeconomic variables into adjusted regression models rather than using interaction terms?

We are using interaction terms specifically for the prespecified subgroup analyses to examine impact heterogeneity in pre-specified subgroups (one of the secondary outcomes), so we would not want to adjust for these covariates here.

Ethics and dissemination:

23. Please provide reference numbers for ethical approvals.

We now provide the reference numbers for the ethical approvals as shown in the text below (lines 416- 419):

"The protocol was reviewed and approved by the Kenya Medical Research Institute (KEMRI) Scientific Ethics Review Unit (SERU) [05/06/424/4632], The Kenya National Commission for Science Technology and Innovation (NACOSTI) Review Board [NACOSTI/P/21/9594], and The London School of Hygiene & Tropical Medicine (LSHTM) Ethics Review Committee [28307]."

24. Please describe consenting procedures for illiterate participants.

We have now added a sentence on the procedure for illiterate participants in lines 424-426:

"*Participants who are unable to sign, apply a thumbprint using an inkpad, having identified a literate impartial witness who sits through the consenting process and additionally signs their own signature on the consent form.*"

1. Legge H, Kazungo K, Muli S, et al. Identifying Potential Determinants of Faecal Contamination on Domestic Floors in Three Settings in Rural Kenya: A Mixed Methods Analysis. *Environ Health Insights* 2024;18:11786302241246454. doi: 10.1177/11786302241246454 [published Online First: 2024/05/13]
2. Kepha S, Legge H, Halliday KE, et al. Better floors, better health: A theory of change for an improved household flooring intervention in rural communities in Kwale and Bungoma counties, Kenya. *BMC Public Health* 2025 doi: 10.1186/s12889-025-21469-1
3. Elson L, Nyawa SM, Matharu A, et al. Developing low-cost house floors to control tungiasis in Kenya - a feasibility study. *BMC Public Health* 2023;23(1):2483. doi: 10.1186/s12889-023-17427-4 [published Online First: 2023/12/13]
4. Cattaneo MD, Galiani S, Gertler PJ, et al. Housing, Health, and Happiness. *American Economic Journal: Economic Policy* 2009;1(1):75-105. doi: DOI: 10.1257/pol.1.1.75
5. Teare J, Mathee A, Naicker N, et al. Dwelling Characteristics Influence Indoor Temperature and May Pose Health Threats in LMICs. *Ann Glob Health* 2020;86(1):91. doi: 10.5334/aogh.2938 [published Online First: 2020/08/25]
6. Grzeskowiak S, Sirgy MJ, Lee D-J, et al. Housing well-being: Developing and validating a measure. *Social Indicators Research* 2006;79:503-41.
7. Thomson H, Thomas S. Developing empirically supported theories of change for housing investment and health. *Social Science & Medicine* 2015;124:205-14.
8. Feldmeier H, Heukelbach J, Ugbomoiko US, et al. Tungiasis--a neglected disease with many challenges for global public health. *PLoS Negl Trop Dis* 2014;8(10):e3133. doi: 10.1371/journal.pntd.0003133 [published Online First: 2014/10/31]
9. Murray M, Versteeg M, Hugo J. The impact of diarrhoea in infants on the quality of life of low-income households. *South African Family Practice* 2008;50(2):1.
10. The global problem of insufficient sleep and its serious public health implications. Healthcare; 2018. MDPI.
11. Elson L, Kamau C, Koech S, et al. National prevalence and risk factors for tungiasis in Kenya. *Infect Dis Poverty* 2023;12(1):85. doi: 10.1186/s40249-023-01131-x [published Online First: 2023/09/19]
12. Elson L, Matharu AK, Riithi N, et al. Characterization of tungiasis infection and morbidity using thermography in Kenya revealed higher disease burden during COVID-19 school closures. *Infect Dis Poverty* 2023;12(1):24. doi: 10.1186/s40249-023-01080-5 [published Online First: 2023/03/22]
13. Pullan RL, Halliday KE, Oswald WE, et al. Effects, equity, and cost of school-based and community-wide treatment strategies for soil-transmitted helminths in Kenya: a cluster- randomised controlled trial. *The Lancet* 2019;393(10185):2039-50.

14. KNBS and ICF. Kenya Demographic and Health Survey 2022. Children's Dataset. Nairobi, Kenya, and Rockville, Maryland, USA: KNBS and ICF, 2023.
15. KNBS and ICF. Kenya Demographic and Health Survey 2022. Key Indicators Report. Nairobi, Kenya, and Rockville, Maryland, USA: KNBS and ICF, 2023.
16. Mwai J, Nyole D, Abdi MH, et al. Factors associated with tungiasis among school-age children in Kwale County, rural Kenya. *Int Health* 2023;15(1):85-92. doi: 10.1093/inthealth/ihac013 [published Online First: 2022/04/08]
17. Mwandawiro C, Okoyo C, Kihara J, et al. Results of a national school-based deworming programme on soil-transmitted helminths infections and schistosomiasis in Kenya: 2012–2017. *Parasites & vectors* 2019;12(1):76.
18. Dangour AD, Watson L, Cumming O, et al. Interventions to improve water quality and supply, sanitation and hygiene practices, and their effects on the nutritional status of children. *Cochrane Database Syst Rev* 2013;2013(8):Cd009382. doi: 10.1002/14651858.CD009382.pub2 [published Online First: 2013/08/02]
19. Ayana M, Cools P, Mekonnen Z, et al. Comparison of four DNA extraction and three preservation protocols for the molecular detection and quantification of soil-transmitted helminths in stool. *PLoS Negl Trop Dis* 2019;13(10):e0007778. doi: 10.1371/journal.pntd.0007778 [published Online First: 2019/10/29]
20. Katz N, Chaves A, Pellegrino J. A simple device for quantitative stool thick-smear technique in Schistosomiasis mansoni. *Rev Inst Med Trop Sao Paulo* 1972;14(6):397-400. [published Online First: 1972/11/01]
21. Child HT, Wierzbicki L, Joslin GR, et al. Comparative evaluation of soil DNA extraction kits for long read metagenomic sequencing. *Access Microbiology* 2024;6(9):000868. v3.

VERSION 2 - REVIEW

Reviewer 1

Name Zirimenya , Ludoviko

Affiliation MRC/UVRI and LSHTM Uganda Research Unit, Immunomodulation and Vaccines Programme

Date 10-Feb-2025

COI

None, all comments have been addressed.
